# Segmentation of Non-Small Cell Lung Carcinomas: Introducing DRU-Net and Multi-Lens Distortion

**DOI:** 10.3390/jimaging11050166

**Published:** 2025-05-20

**Authors:** Soroush Oskouei, Marit Valla, André Pedersen, Erik Smistad, Vibeke Grotnes Dale, Maren Høibø, Sissel Gyrid Freim Wahl, Mats Dehli Haugum, Thomas Langø, Maria Paula Ramnefjell, Lars Andreas Akslen, Gabriel Kiss, Hanne Sorger

**Affiliations:** 1 Department of Circulation and Medical Imaging, Norwegian University of Science and Technology (NTNU), NO-7491 Trondheim, Norway; 2 Clinic of Medicine, Levanger Hospital, Nord-Trøndelag Health Trust, NO-7600 Levanger, Norway; 3 Department of Clinical and Molecular Medicine, Norwegian University of Science and Technology (NTNU), NO-7491 Trondheim, Norway; 4 Clinic of Laboratory Medicine, St. Olavs Hospital, Trondheim University Hospital, NO-7030 Trondheim, Norway; 5 Clinic of Surgery, St. Olavs Hospital, Trondheim University Hospital, NO-7030 Trondheim, Norway; 6 Application Solutions, Sopra Steria, NO-7010 Trondheim, Norway; 7 Department of Health Research, SINTEF Digital, NO-7465 Trondheim, Norway; 8 Department of Pathology, St. Olavs Hospital, Trondheim University Hospital, NO-7030 Trondheim, Norway; 9 Center for Innovation, Medical Devices and Technology, Research Department, St. Olavs Hospital, Trondheim University Hospital, NO-7491 Trondheim, Norway; 10 Centre for Cancer Biomarkers CCBIO, Department of Clinical Medicine, University of Bergen, NO-5007 Bergen, Norway; 11 Department of Pathology, Haukeland University Hospital, NO-5020 Bergen, Norway; 12 Department of Computer Science, Norwegian University of Science and Technology (NTNU), NO-7491 Trondheim, Norway

**Keywords:** lung carcinoma, digital pathology, tumor segmentation, deep learning, data augmentation

## Abstract

The increased workload in pathology laboratories today means automated tools such as artificial intelligence models can be useful, helping pathologists with their tasks. In this paper, we propose a segmentation model (DRU-Net) that can provide a delineation of human non-small cell lung carcinomas and an augmentation method that can improve classification results. The proposed model is a fused combination of truncated pre-trained DenseNet201 and ResNet101V2 as a patch-wise classifier, followed by a lightweight U-Net as a refinement model. Two datasets (Norwegian Lung Cancer Biobank and Haukeland University Lung Cancer cohort) were used to develop the model. The DRU-Net model achieved an average of 0.91 Dice similarity coefficient. The proposed spatial augmentation method (multi-lens distortion) improved the Dice similarity coefficient from 0.88 to 0.91. Our findings show that selecting image patches that specifically include regions of interest leads to better results for the patch-wise classifier compared to other sampling methods. A qualitative analysis by pathology experts showed that the DRU-Net model was generally successful in tumor detection. Results in the test set showed some areas of false-positive and false-negative segmentation in the periphery, particularly in tumors with inflammatory and reactive changes. In summary, the presented DRU-Net model demonstrated the best performance on the segmentation task, and the proposed augmentation technique proved to improve the results.

## 1. Introduction

Early diagnosis of lung cancer is crucial for patient survival [1]. Although physical examinations and medical imaging are included in the diagnostic work-up, tissue samples are needed to establish a cancer diagnosis. The histopathological diagnosis, including the analysis of tumor biomarkers, influences therapeutic decisions and should, therefore, be assessed as early and accurately as possible [2,3].

Digitizing tissue slides allows evaluation via computer screens, which can improve efficiency over traditional microscopy [4]. It also supports AI-driven tissue classification, segmentation, potentially increasing the speed of image interpretation, and refining clinical decision-making [5,6,7,8]. Correct segmentation of the tumor is a necessary step towards computer-assisted tumor analysis and lung cancer diagnosis [9,10,11,12,13,14].

When working with whole slide images (WSIs), the application of AI models is complicated due to the large size of the images. Down-sampling the WSIs to a manageable size would compromise resolution and potentially result in the loss of critical diagnostic details. A common approach in digital pathology is, therefore, to divide the images into several small squares, called patches. This is a more effective approach, but the use of patch-based analysis alone can lead to a loss of broader spatial relationships. Alternatively, the image can be down-sampled, or a hybrid strategy that combines both methods can be used to optimize the analytical balance between detailed resolution and global context.

Some of the best-performing AI methods in the analysis of WSIs are deep neural networks [14,15]. The state-of-the-art in image segmentation tasks is the use of complex neural network architectures such as vision transformers and InternImage [16,17]. However, these methods require a relatively large amount of data [18]. Transfer learning techniques may also be used to train or fine-tune pre-trained models on new data [19]. Patch-wise classification (PWC) or segmentation approaches may outperform direct segmentation of the tumor in a down-sampled image without dividing it into patches [20].

Several models have been proposed for tumor segmentation in WSIs [11,21,22,23,24,25,26,27,28,29]. Zhao et al. proposed a novel hybrid deep learning framework for colorectal cancer that uses a U-Net architecture. This model features innovative residual ghost blocks, which include switchable normalization and bottleneck transformers for extracting features [11].

The MAMC-Net model introduced a multi-resolution attention module that utilizes pyramid inputs for broader feature information and detail capture [21]. An attention mechanism refines features for segmentation, while a multi-scale convolution module integrates semantic and high-resolution details. Finally, a connected conditional random field ensures accurate segmentation by addressing discontinuities [21]. The authors showcased the superior performance of their model on breast cancer metastases and gastric cancer [21].

DHU-Net combines Swin Transformer and ConvNeXt within a dual-branch hierarchical U-shaped architecture [22,30,31]. This method effectively fuses global and local features by processing WSI patches through parallel encoders, utilizing global-local fusion modules and skip connections for detailed feature integration [22]. The Cross-scale Expand Layer aids in resolution recovery across different scales. The network was evaluated on datasets covering different tumor features and cancer types, and achieved higher segmentation results than other tested methods [22].

Krikid et al. showed that deep-learning applications in microscopic image segmentation have evolved from predominantly cell- and nucleus-centric tasks—often on small, homogeneous datasets—to encompass more complex, tissue-level analyses, reflecting a shift toward multi-scale, clinically relevant segmentation across diverse microscopy-modality types [32]; Greeley et al. introduced pyramid tiling for efficient gigapixel histology analysis [33]; promptable models like SAM and MedSAM enable zero-shot, universal segmentation across modalities [34,35].

Pedersen et al. introduced H2G-Net, a cascaded convolutional neural network (CNN) architecture for segmenting breast cancer regions from gigapixel histopathological images [23]. It employs a patch-wise detection stage and a convolutional autoencoder for refinement, demonstrating significant improvements in tumor segmentation. The approach outperformed single-resolution methods, achieving a Dice similarity coefficient (DSC) of (0.933 ± 0.069) [23]. Its efficiency is underscored by fast processing times and the ability to train deep neural networks without having to store patches on disk.

One of the most significant challenges in using WSIs for tumor segmentation is still the scarcity of labeled data. The marking of tumor tissue in WSIs by pathology experts is time-consuming and may be a bottleneck in research. Alternative computational strategies, such as unsupervised or semi-supervised learning methods should, therefore, be explored. Clustering allows the segmentation of tumor regions with little or no need for predefined labels, and can be a useful tool in this context [24,25].

Yan et al. presented a self-supervised learning method using contrastive learning to process WSIs for tissue clustering [26]. This approach generates discriminative embeddings for initial clustering, refined by a silhouette-based scheme, and extracts features using a multi-scale encoder [26]. It achieved high accuracy in identifying tissues without annotations. Their results show an area under the curve (AUC) of 0.99 and accuracy of approximately 0.93 for distinguishing benign from malignant polyps in a cohort of 20 patients [26].

Few-shot learning is also a promising method for handling limited labeled data [27,28]. By design, few-shot learning algorithms can learn from a very limited number of labeled examples. This can be particularly relevant for the classification of small patches, where a small set of labeled examples can guide the learning process. Few-shot learning techniques can generalize from these examples to classify new, unseen patches, facilitating the identification and segmentation of tumor regions [27,28]. Titoriya et al. explored few-shot learning to enhance dataset generalization and manageability by utilizing prototypical networks and model agnostic meta-learning across four datasets [29]. The design achieved 85% accuracy in a 2-way 2-shot 2-query mode [29].

In this paper, we propose a new CNN-based model which is a combination of DenseNet [36], ResNet [37], and U-Net architecture (DRU-Net) for segmenting non-small cell lung carcinomas (NSCLCs). It is an end-to-end approach consisting of a dual head for feature extraction and patch classification, followed by a U-Net for refining the segmentation result. The proposed model is tested on a novel in-house dataset of 97 annotated NSCLC WSIs. To increase model performance, we adopted a many-shot learning approach during training and added a multi-lens distortion augmentation technique to both patches and down-sampled WSIs.

## 2. Materials and Methods

### 2.1. Cohorts

In this study, two different collections of NSCLCs were used: the Norwegian lung cancer biobank (NLCB) cohort and Haukeland University lung cancer (HULC) cohort [38,39]. The NLCB cohort includes histopathological, cytological, biomarker, and clinical follow-up data from patients with suspected lung cancer diagnosed in Central Norway after 2006 [40]. Both diagnostic tumor biopsies and sections from surgical lung cancer specimens are available. The distribution of histological subtypes in each dataset is listed in Table 1 [41,42].

The HULC cohort comprises 438 surgically treated NSCLC patients diagnosed at Haukeland University Hospital, Bergen, Norway from 1993 to 2010. In this study, 97 NSCLC cases from the HULC cohort were included. From both cohorts, 4 µm tissue sections were made, deparaffinized, rehydrated in ethanol, and immersed in tap water. Hematoxylin staining was applied and sections were rinsed in water and then in ethanol. Sections were then stained with alcoholic eosin. Post-staining, slides were dehydrated in ethanol, placed in TissueClear, air-dried, and scanned using Olympus VS120-S5 scanner (Olympus Soft Imaging Solutions GmbH, Munster, Germany) at ×40 magnification [43]. WSIs were quality-controlled by a pathologist to ensure that only high-quality scans were included in the study. They were reviewed for sectioning, staining, and scanning artifacts.

To conduct a broader study of the proposed augmentation’s effect, we utilized the following open datasets in addition to HULC: MNIST, Fashion-MNIST, CIFAR-10, and CIFAR-100 [44,45,46].

### 2.2. Ethical Aspects

All methods were carried out in accordance with relevant guidelines and regulations, and the experimental protocols were approved by the Regional Committee for Medical and Health Sciences Research Ethics (REK) Norway (2013/529, 2016/1156, and 257624). Informed consent was obtained from all subjects and/or their legal guardian(s) for NLCB in accordance with REK 2016/1156. For subjects in the HULC cohort, exemption from consent was ethically approved by REK (2013/529).

### 2.3. Annotations and Dataset Preparation

We used two annotation approaches on WSIs: whole tumor annotation (WTA) and partial selective annotation (PSA). In the WTA approach, pathologists marked the tumor outline in 97 WSIs from the HULC cohort. Of these WSIs, 51 were used for training, 26 were used for validation, and 20 were used for testing. WSIs with tissue microarray (TMA) holes (*n* = 3) were manually assigned to the test set to prevent potential biased training; the remaining WSIs were randomly separated into the training, validation, and the rest of the test sets.

To reduce the time spent by pathologists in making the WTAs, initial annotations were first made in 72 cases using two different AI-based segmentation models, (i) the H2G-Net model developed for breast cancer segmentation (*n* = 25) and (ii) a customized early-stage clustering model based on the corrected annotations from the H2G-Net model (*n* = 47) [23]. Pathologists then manually refined the tumor region annotations using the QuPath software (version 0.3.2) [47]. The remaining 25 cases were manually annotated without any prior AI-based segmentation models. A third pathologist reviewed the annotations, and in case of discrepancy, consensus was reached after discussion. The final annotations were exported as binary masks, serving as ground truth.

In the PSA approach, pathologists marked small regions of interest in 42 WSIs from the NLCB cohort. These WSIs were used for training and validation of the patch-wise classifier model. Marked areas included parts of the invasive tumor, normal alveolar tissue, stromal tissue, immune cells, and areas of necrosis. Other non-tumor tissues marked included respiratory epithelium, reactive alveolar tissue, cartilage, blood vessels, glands, lymph nodes, and macrophages. The purpose of marking these regions was to reduce the time required for manually annotating whole tumor regions, and to guide a particular selective generation of patches intended for use in the patch-wise model’s training.

### 2.4. Proposed Method

The pipeline of the proposed model (DRU-Net) has two distinct stages, a PWC stage and a refinement stage. The PWC model was trained on the NLCB cohort using a many-shot learning method, and the refinement U-Net was trained on a set of down-sampled WSIs from the HULC cohort. In the PWC stage, the model assigns probabilities to each patch of the WSIs (excluding the glass), indicating whether the patch contains tumor tissue or non-tumor tissue. The classifier outputs a preliminary assessment of each patch’s nature, based on local features within the patch. The patches are then stitched together to produce a heatmap matching the original size of the down-sampled WSIs.

#### 2.4.1. Patch-Wise Classifier

**Figure 1:**
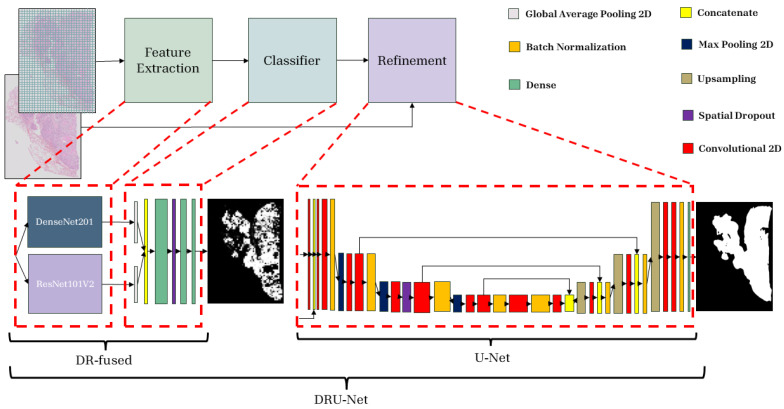
Illustration of the proposed DRU-Net model. The patched image is fed into the classifier part. The output of the classifier is combined with a down-sampled WSI as an input for the refinement head.

The PWC was constructed by fusing truncated backbones of two architectures, DenseNet201 [36] and ResNet101V2 [37], pre-trained on ImageNet [48]. We conducted a preliminary search on a dataset subset to determine the most effective truncation points for both DenseNet and ResNet backbones. This empirical exploration guided our layer selection based on performance. These networks are used for parallel processing of the input and feature generation (we refer to this PWC model as DR-Fused). In our proposed architecture, both DenseNet201 and ResNet101V2 receive the same input, which is the image patch. Each network processes this input concurrently, and after feature extraction, the outputs from both DenseNet201 and ResNet101V2 pass through their respective global average pooling layers. This step compresses the feature representation to help prevent overfitting. The compressed features from both networks are then concatenated and fed through the classifier head (Figure 1).

#### 2.4.2. Refinement Network

The heatmap is generated from applying the PWC across the WSIs. The resultant heatmap is then resized and concatenated with a down-sampled version of the WSI (1120×1120 pixels). The fused inputs are then fed to a refinement network, similar to H2G-Net [23]. Using a refinement network allows for adjusting the initial patch-wise predictions based on global WSI-level information.

The proposed refinement network is a simple, lightweight U-Net architecture, specifically tailored to process two image inputs (Figure 1). In this model, the two inputs (down-sampled RGB WSI and the heatmap) are concatenated into a 4-channel image and then processed through multiple convolutional layers with ReLU activation functions, batch normalization, spatial dropout, skip connections, max pooling, and up-sampling layers (with nearest-neighbor interpolation). The network ends with a softmax activation function.

#### 2.4.3. Data Augmentation

To improve model robustness, data augmentation is commonly performed. Data augmentation generates artificial copies of the training data through a predefined algorithm. This allows the training data to better cover the expected data variation. Data augmentation was integrated into the training data generation process, with the following methods applied randomly: vertical and horizontal flipping, rotations (multiples of $90^{{}^{\circ}}$), multiplicative contrast adjustment, hue and brightness variations, and the proposed multi-lens distortion augmentation. During the many-shot learning using PSA, we extracted patches by cropping a random 224×224-pixel section from each image. Each image appeared only once per epoch, where an epoch is defined as one iteration of all the training data.

#### 2.4.4. Multi-Lens Distortion Augmentation

A novel data augmentation method, multi-lens distortion, was developed to simulate several local random lens distortions. This technique aims to allow the model to recognize the important features of the images under a wider range of cell/tissue shapes.

**Figure 2:**
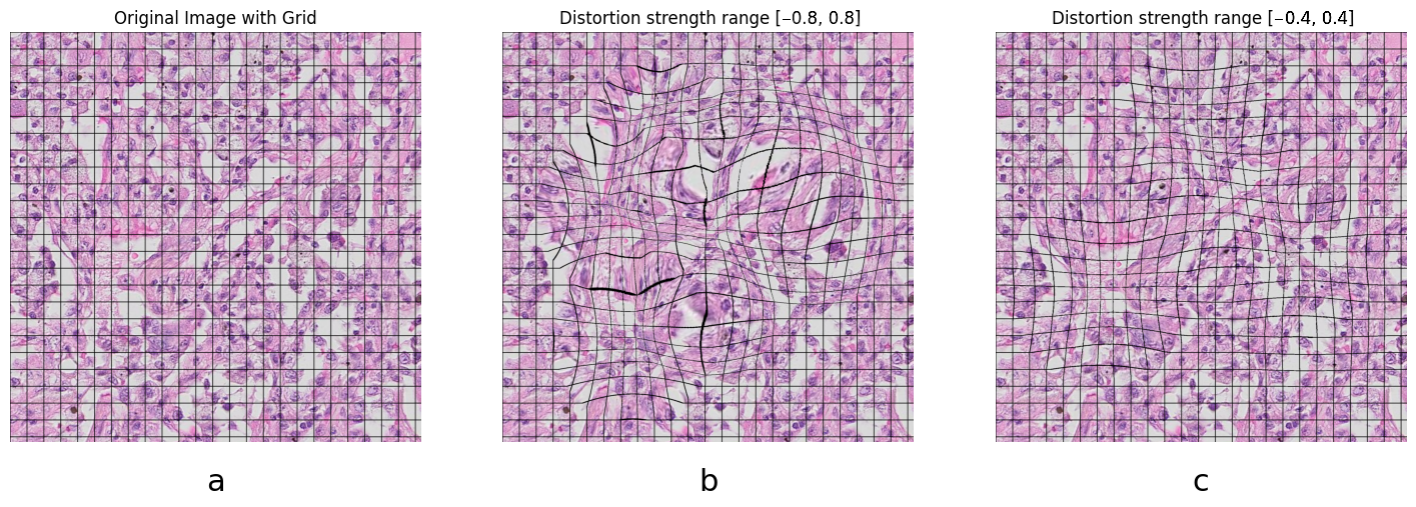
Sample effect of the novel augmentation on a patch with overlaid grids to illustrate the effect. (**a**) Original image showing epithelial cells. (**b**) Augmented image with parameters set too high, cell size variation and deformation are visible. (**c**) Augmented image with a medium setting of the parameters.

The algorithm uses a predefined number of lenses. For each lens, a random position within the image is selected. Then, a random distortion radius and strength value are used to apply the barrel and/or pincushion distortion effect at the selected positions (Algorithm 1). An example of this augmentation is shown in Figure 2. The optimal radius range and lens count were established empirically through an iterative series of experiments, with each configuration assessed qualitatively to identify the most compelling results. From a histopathology point of view, too strong augmentations produce morphologically invalid images, which degrade performance. Thus, it is necessary to specifically tune these parameters for the targeted applications, especially in healthcare.
**Algorithm 1** Multi-Lens Distortion (implementation-level pseudocode)**Require:** $img\in R^{H\times W\times C}$, *N*   ▹ number of lenses, $\left(r_{\min},r_{\max}\right)$, $\left(s_{\min},s_{\max}\right)$ **Ensure:** $out\in R^{H\times W\times C}$
 1: out←img                       ▹ deep copy 2: (yidx,xidx)←meshgrid(0:H−1,0:W−1) 3:  **for** i←1 **to** *N* **do** 4:         cx←randInt(0,W−1) 5:         cy←randInt(0,H−1) 6:         $R\leftarrow \mathrm{randInt}(r_{\min},\;r_{\max})$ 7:         $S\leftarrow \mathrm{randFloat}(s_{\min},\;s_{\max})$ 8:         **for all** (y,x) **in** {0:H−1}×{0:W−1} **do** 9:               dx←x−cx;dy←y−cy10:              $r\leftarrow \sqrt{dx^{2}+dy^{2}}$11:              **if** r<R **then**12:                   $\hat{r}\leftarrow r/R$             ▹ normalised distance13:                   $sf\leftarrow 1-\hat{r}$               ▹ scaling factor14:                   scale←1−S·sf15:                   $x_{new}\leftarrow cx+dx\cdot scale$16:                   $y_{new}\leftarrow cy+dy\cdot scale$17:                   $x_{new}\leftarrow \mathrm{clamp}\left(x_{new},\;0,\;W-1\right)$18:                   $y_{new}\leftarrow \mathrm{clamp}\left(y_{new},\;0,\;H-1\right)$19:                   $out\left[y,x\right]\leftarrow img\left[y_{new},\;x_{new}\right]$20:              **end if**21:         **end for**22:  **end for**23:  **return** out


#### 2.4.5. Model Training

The PWC network was fine-tuned to adapt to the specific task by freezing the initial layers. The following training parameters were included: optimizer: Adamax with a learning rate of $1\times 10^{-4}$; loss function: categorical crossentropy; metrics: F_1_-score; batch size: dynamically determined based on the training generator configuration; epochs: up to 200 with early stopping based on validation loss to prevent overfitting.

The refinement network training involved the following: optimizer: Adam with a learning rate of $1\times 10^{-4}$; loss function: Dice loss function, optimized for segmentation tasks; metrics: Thresholded Dice score; batch size: 2; epochs: up to 300 with early stopping based on validation loss to prevent overfitting; training environment: utilization of GPU and memory growth settings to optimize hardware usage.

In the WTA method, the same set of slides was used for both PWC and segmentation models’ training. From the 97 slides, 77 slides were randomly chosen and divided into training and validation sets in a 2:1 ratio, with 51 and 26 slides, respectively, while 20 slides (including those with TMA holes) were used for testing.

WSIs in the dataset from the HULC cohort were divided into tiles (patches) and each tile was fed into the neural network along with the non-tumor/tumor label based on the provided annotation. To create the annotation labels for patches, non-tumor and tumor tiles were assigned the values 0 and 1, respectively. We first used a threshold on color gradients to separate the tissue from the background glass. Any tile that did not include more than 25% tissue was disregarded, meaning that all the input tiles contained less than 75% background glass. Also, a minimum of 5% of the tumor area was required for a tile to be classified as tumor, and for the non-tumor regions, only tiles with no tumor were assigned. Tiles containing less than 5% tumor area were excluded.

Using the annotated WSI regions with PSA in the NLCB dataset, 40 areas were assigned to the tumor class (labeled as 1) and 50 areas to the non-tumor class (labeled as 0). The selected areas led to the generation of patches in subsequent steps. Specifically, out of 50 areas categorized as non-tumor, 40 clearly lacked tumor characteristics, and 10 showed features slightly above the initial threshold, as shown in Supplementary Figure S2. This threshold was established through model training before intentionally creating an imbalance in the dataset. The imbalance was introduced after unsuccessful attempts to enhance model generalizability through various methods, including weighted loss functions, focal loss, threshold adjustment, and sampling strategies.

#### 2.4.6. Post-Processing

After the segmentation results were received, two post-processing steps were performed. First, small fragments were removed by converting images into grayscale and then to binary format to identify and eliminate fragments smaller than a fixed threshold. The threshold was set to the smallest annotated segmentation area in the ground truth. In the second step, an edge smoothing algorithm was applied to enhance image quality. This improvement was achieved through mathematical techniques known as morphological operations, which are commonly used in digital image processing to modify the geometrical structure of images. Specifically, we used a process called morphological opening, which involves an erosion operation followed by a dilation. This sequence helps reduce jagged edges and smooths the boundaries of objects within the image. The operations were performed using a kernel size of 7×7. Additionally, a median blur with a kernel size of 11×11 was applied to further smooth the edges. It is important to note that these morphological operations refer to image processing techniques. They are purely computational methods used to process the digital images and should not be confused with the morphological study of biological tissues.

### 2.5. Implementation

Implementation was conducted in Python 3.8.10. TensorFlow (v2.13.1) was used for model architecture implementation and training [49]. These additional libraries were used for the experiments: pyFAST, OpenCV, NumPy, Pillow, SciPy, scikit-learn, and Matplotlib [50,51,52,53,54,55,56,57]. Trained models were converted to the ONNX format using the tf2onnx library [58]. Converted models were then integrated into FastPathology for deployment [59]. FastPathology is an open-source, user-friendly software developed for deep learning-based digital pathology that offers tools for processing and visualizing WSIs. The source code used to conduct the experiments is made openly available at. https://github.com/AICAN-Research/DRU-Net (accessed on 29 April 2024).

### 2.6. Experiments

To compare the proposed model (DRU-Net) with other models, the following experiments were carried out: modifications of the previously introduced H2G-Net model on both datasets, DRU-Net with the backbone trained on the HULC cohort and NLCB, and applying the few-shot and many-shot learning techniques along with clustering (Table 2) [23].

H2G-Net could be tested as is, and be fine-tuned with five different modifications [23]. First, H2G-Net was tested without any modification, fine-tuning, or additional training, to see whether a model trained for breast cancer tumor delineation can also work for lung cancer. Second, the PWC of the H2G-Net was fine-tuned on annotated WSIs from the HULC cohort, and the original U-Net of H2G-Net was applied on top of the PWC results. Third, the whole model (PWC and U-Net) was fine-tuned on the training data. Then, the same three methods were tested, but with the PWC trained on NLCB instead of the HULC cohort.

An ablation study was performed to evaluate the effect of the proposed multi-lens distortion augmentation. A pre-trained DenseNet121 was tested on four open datasets: MNIST, Fashion-MNIST, CIFAR-10, and CIFAR-100 [44,45,46]. Experiments were repeated with and without this augmentation on the mentioned open datasets by randomly selecting 10% of the training data and the results were compared using Wilcoxon test. Both control and test groups included other augmentation techniques, such as color adjustments, flipping, rotation, brightness, and contrast augmentations. The effect of this augmentation on the training time was measured using the integrated TensorFlow functions by comparing the time with and without the augmentation and the results were averaged on WSIs and compared between the two [49].

We also investigated the effect of removing the top-most skip connection of the U-Net refinement model and we calculated the average Hausdorff distances (HDs) for two sets of final segmentation predictions in comparison to a ground truth set. This was conducted to quantify the effect of removing that skip connection, which was implemented to reduce the small fragments around the segmentation perimeter.

### 2.7. Model Evaluation

#### 2.7.1. Quantitative Model Assessment

To quantitatively validate the patch-wise classification performance, precision, recall, and F_1_-score were used [61]. The validation of the final segmentation on WSI-level was performed using DSC and HD [62].

#### 2.7.2. Qualitative Model Assessment

The qualitative assessment of the segmentation results was conducted by two pathologists using the scoring system described in Table 3. Qualitative assessment was conducted on the same 20 WSIs of the test set from the HULC cohort.

#### 2.7.3. Saliency Maps

To survey the model’s decision-making process and the areas of patches that were most relevant for predicting the tumor class, we employed a method known as gradient-based saliency maps [63,64,65,66]. This approach operates by computing the gradient of the output class (the class for which we want to understand model sensitivity) with respect to the input image. These gradients indicate the sensitivity of the output to each pixel in the input image. By highlighting the pixels with the highest gradients, we can visualize the areas that most strongly influenced the model’s classification decision. We used six different patches selected from six different WSIs from the HULC cohort to analyze the saliency maps. Patches were chosen to represent true positive, false positive, and false negative predictions. Patches with true positive predictions were selected to include various histological features and cell types in each patch to better assess the model’s decision process.

#### 2.7.4. Computation of FLOPs and Parameters

To quantitatively assess the computational complexity and model size, we calculated the number of floating-point operations (FLOPs) and the total number of trainable parameters for all evaluated models, including DR-Fused and several standard architectures. For each model, FLOPs were estimated by converting the model into a frozen computational graph using TensorFlow’s convert_variables_to_constants_v2 function, followed by profiling with tf.compat.v1.profiler. The FLOPs represent the total number of arithmetic operations required for a single forward pass of an input image sized 224×224×3. Parameter counts were obtained directly via the count_params method provided by TensorFlow. All FLOPs and parameter values were reported in millions (M) for clarity. MobileNetV2 was designated as the baseline model. Relative changes in FLOPs and parameters (ΔFLOPs and ΔParams) were computed for each model compared to MobileNetV2, using the following formulas:

(1) $$\Delta \mathrm{FLOPs}\;\left(\%\right)=\frac{{\mathrm{FLOPs}}_{\mathrm{model}}-{\mathrm{FLOPs}}_{\mathrm{baseline}}}{{\mathrm{FLOPs}}_{\mathrm{baseline}}}\times 100$$

(2) $$\Delta \mathrm{Params}\;\left(\%\right)=\frac{{\mathrm{Params}}_{\mathrm{model}}-{\mathrm{Params}}_{\mathrm{baseline}}}{{\mathrm{Params}}_{\mathrm{baseline}}}\times 100$$

## 3. Results

**Figure 3:**
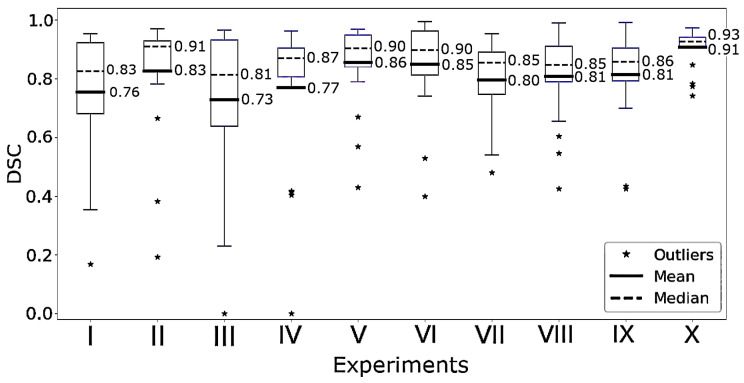
Boxplots of the Dice similarity coefficients (DSCs) of the experiments shown Table 2 on the 20 WSIs of the test set. (I) original H2G-Net, (II) H2G-Net with fine-tuned PWC on HULC cohort, (III) H2G-Net with fine-tuned U-Net on HULC cohort, (IV) H2G-Net with fine-tuned PWC and U-Net on HULC cohort, (V) DRU-Net trained on HULC Cohort, (VI) H2G-Net with fine-tuned PWC on NLCB, (VII) H2G-Net with fine-tuned PWC on NLCB and fine-tuned U-Net on HULC Cohort, (VIII) FSC, (IX) MSC, (X) DRU-Net with PWC trained on NLCB and U-Net trained on HULC Cohort.

**Figure 4:**
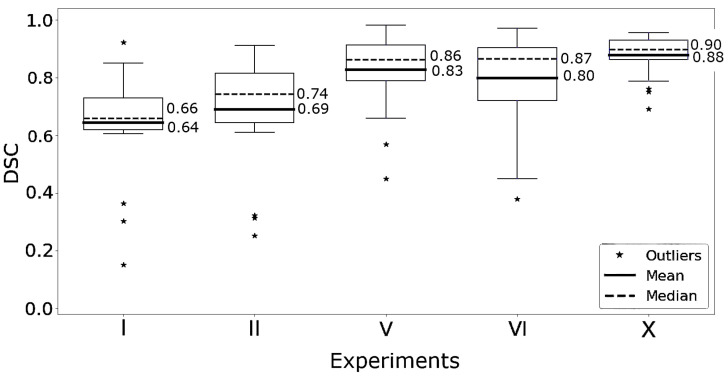
Boxplot of the Dice similarity coefficients (DSCs) of the PWC models in experiments listed in Table 2 without the refinement network, only the patch-wise classifier is used to produce these results. (I) original H2G-Net, (II) H2G-Net with fine-tuned PWC on HULC cohort, (V) DR-Fused trained on HULC Cohort, (VI) H2G-Net with fine-tuned PWC on NLCB, (X) DR-Fused trained on NLCB and U-Net trained on HULC Cohort.

The highest DSC on average on the 20 WSIs of the test set from HULC cohort was achieved by DRU-Net, followed by the H2G-Net with fine-tuned PWC on the HULC cohort (Figure 3). Similar differences in DSC were observed for the models without the refinement networks (Figure 4).

**Figure 5:**
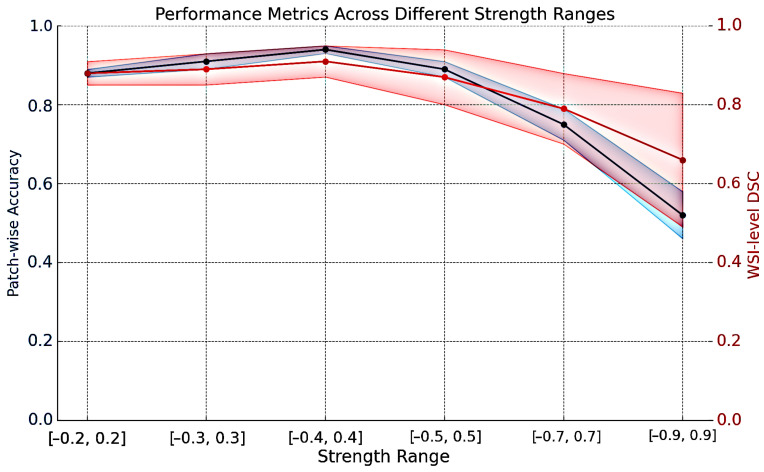
The impact of the multi-lens distortion augmentation technique using the DRU-Net model. DSC: Dice similarity coefficient. The highlighted regions indicate the variance, and the mean values are shown on the curve.

Proposed multi-lens distortion augmentation applied to various datasets resulted in increased F_1_-score overall, this change was statistically significant when applied to our dataset from the NLCB (Table 4). Applying this augmentation technique increased training time by an average of 8%. DSC and patch-wise accuracy increased when multi-lens distortion augmentation was used with a magnitude strength in the range [−0.4, 0.4], but higher magnitudes caused a decrease in performance (Figure 5).

The original H2G-Net resulted in an average of 0.76 DSC (Figure 3) and 0.66 intersection over union (IOU) scores. On average, 25% of the non-tumor regions around the true tumor outlines were falsely labeled as tumor. When the PWC component of the model was used without refinement, the predictions resulted in 0.64 DSC and 0.61 IOU, showing that the refinement improved the predictions significantly.

A fine-tuned PWC trained and validated on 77 WSIs from the HULC cohort, with the direct implementation of the pre-trained U-Net from H2G-Net, was tested on 20 WSIs from the HULC cohort and resulted in an average of 0.83 DSC (median 0.91) (Figure 3) and an average of 0.74 IOU scores. Scores were reduced to an average of 0.77 DSC (median of 0.87) and an average of 0.69 IOU when both the U-Net and the PWC were fine-tuned.

**Figure 6:**
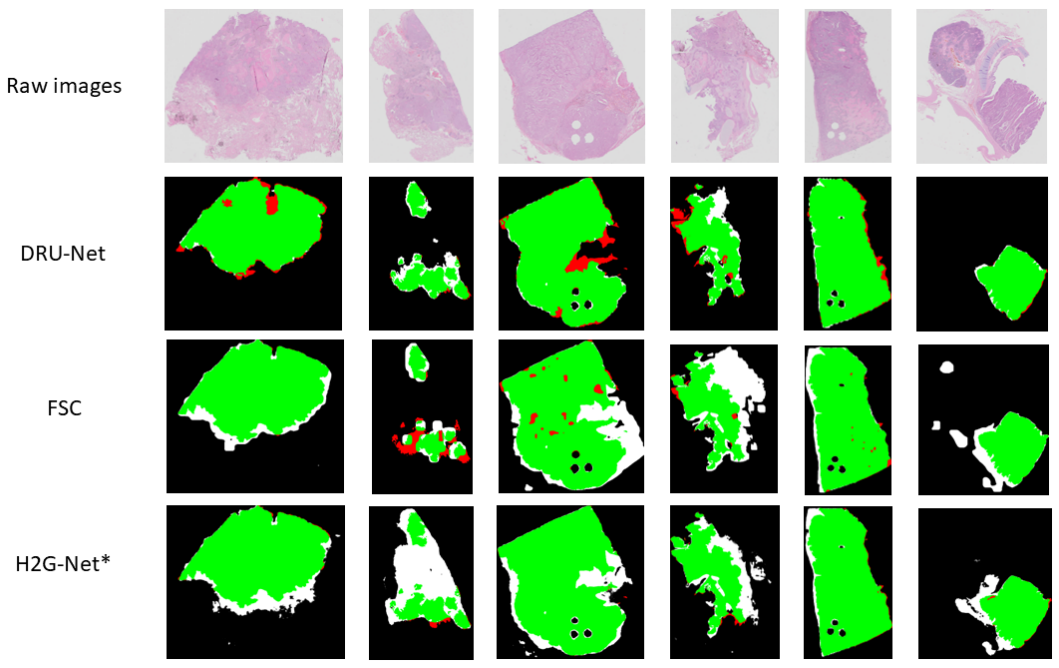
Sample results of three tested networks. First row: original whole slide images (WSIs), second row: DRU-Net, third row: FSC (Few-shot learning + clustering), fourth row: H2G-Net with fine-tuned patch-wise classifier and original U-Net. Green pixels indicate true positives, White pixels indicate false positives and red pixels indicate false negatives. * Indicates that this is not the original H2G-Net, but a modified version.

The proposed model (DRU-Net) tested on the same 20 WSIs resulted in an average of 0.91 DSC (median 0.93) and 0.81 IOU. Also, removing the top skip connection in our U-Net model (DRU-Net) resulted in an average reduction in HD by 4.8%. Figure 6 shows a comparison of the results from various models. Table 5 summarizes various backbones’ performance in the patch-wise classifier component of the model.

In addition to the classification performance, we evaluated the computational complexity of each backbone in terms of FLOPs (floating point operations) and number of parameters, as summarized in Table 6. While the proposed DR-Fused backbone exhibits higher computational cost compared to lightweight models such as MobileNetV2 [60], it remains significantly more efficient than very large networks like VGG19 [67] and ResNet101V2 [37]. Importantly, the DR-Fused model achieves substantial improvements in classification performance (Table 5), with an F_1_-score of 0.94 compared to 0.86 for MobileNetV2 and 0.91 for DenseNet201 [36].

We compared the performance of several models on processing a set of 20 WSIs, with the average dimensions being approximately 108,640 pixels in width and 129,835 pixels in height. H2G-Net and its fine-tuned versions were the fastest models during inference (62 s). Although the many-shot and few-shot models had faster training, they exhibited slower runtimes, with MSC taking the longest at 167 s and DRU-Net at 152 s.

**Figure 7:**
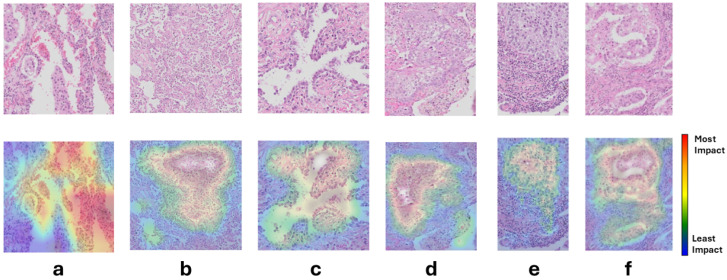
Sample patches (**top** row) and their overlaid saliency maps (**bottom** row); only the patches were given to the PWC model. Note that the saliency map does not indicate malignancy; instead, it shows how different regions of the image influence the classification decision. The colors on the map range from blue, indicating the least influence, to red, which indicates the most influence. (**a**) shows a false negative where it misses the tumor, (**b**,**c**) show false positive tumor detection. (**d**–**f**) show true positive tumor detection. (**a**) shows three small sheets of atypical epithelial tumor cells, of which only one is highlighted in red. The remaining tissue comprises widened alveolar septae with inflammatory cells, pigmented macrophages, reactive pneumocytes and red blood cells. (**b**) includes reactive pneumocytes and macrophages. (**c**) shows reactive pneumocyte hyperplasia. (**d**) presents a solid tumor with enlarged nuclei, where the majority of the model’s focus lies; the peripheral parts of the patch contain alveolar tissue. (**e**) highlights a solid tumor (mostly in yellow) alongside inflammatory cells (primarily in blue). (**f**) shows a solid tumor with areas of necrosis (mainly highlighted in yellow and red) as well as fibrous tissues with inflammatory cells, predominantly marked in blue and some green.

The results of the saliency map analysis in six patches are shown in Figure 7. False-positive areas in the saliency maps were partly explained by areas with reactive pneumocytes, macrophages, and reactive pneumocyte hyperplasia.

The qualitative assessment resulted in an average score of 3.95 out of 5. In nine of the cases assessed, there were sparse areas in the periphery of the tumor that the model misclassified.

## 4. Discussion

In this paper, we introduce a novel deep learning-based model to segment the outline of NSCLCs. We have incorporated a patch-wise classifier, synergistically integrating truncated DenseNet201 [36] and ResNet101V2 [37] architectures, enhanced by a segmentation refinement U-Net model. The proposed composite PWC model demonstrated superior performance over other tested backbones. Due to our relatively small dataset and considering the desired memory and speed efficiency, CNNs were preferred in this study. Using transformer-based models would have required more extensive datasets and computational resources [70,71].

This study also resulted in a novel dataset comprising annotated NSCLCs and marked regions of interest in WSIs from NSCLCs, covering various tissue types. Our results indicate that the PSA approach yielded more effective training outcomes for the patch-wise classifier than the WTA techniques, both with and without class balancing via tissue clustering. Using the WTA approach, annotations were extremely time-consuming for expert pathologists (including review and correction). However, the PSA method significantly reduced this time by an order of magnitude.

Our study demonstrated that the implementation of the multi-lens distortion augmentation technique enhanced classification outcomes across diverse datasets with limited volume of training data. However, the effect of this augmentation could vary depending on the data themselves. We investigated the effect of the augmentation’s strength range on the patch-wise classification accuracy and refinement network’s DSC on WSI-level, concluding that the degree of augmentation is pivotal for its impact on the training process. Excessively strong distortion of images could obstruct the model’s ability to learn relevant patterns, as shown in the impact of the multi-lens distortion augmentation with various strength ranges (Figure 5). It is important to note that the effective range is dependent on the dataset, and the same values may not necessarily yield similar improvements across different datasets.

The non-linear warping introduced by the multi-lens distortion mimics the subtle spatial deformations, slight micro-stretches of the tissue, and local distortions. By applying controlled, spatially varying warps at different scales, our augmentation reproduces these effects. This generates realistic variations in cell and tissue morphology. This not only strengthens model robustness to scanner-induced artifacts, but also promotes generalization across varying magnification levels, shapes, stretches, and similar sample-preparation conditions.

Instead of stain normalization techniques, we used an augmentation-based approach to produce more robustness, maintain important staining details, reduce computational complexity, and safeguard essential characteristics from unintended modification. The dataset already had consistent staining, which eliminated the need for traditional stain normalization [72,73]. To mimic the wide range of HE staining protocols seen across laboratories, we applied the mentioned randomized adjustments in brightness, contrast, and hue during training. By exposing the model to these controlled, biologically plausible variations in color balance and intensity, we effectively simulate batch-to-batch and site-to-site staining differences.

The RGSB-UNet model features a unique hybrid design that combines residual ghost blocks with switchable normalization and a bottleneck transformer [11]. This design focuses on extracting refined features through its complex structure. However, our study found that simpler and more synergistic architectures can also effectively extract reliable features.

The MAMC-Net model improves tumor boundary detection by using a conditional random field layer [21], whereas the DRU-Net model enhances segmentation by fine-tuning a U-Net on a down-sampled image. While both methods achieved good results, our approach—using a U-Net on down-sampled images—proved faster and highly efficient. Notably, our model using the PSA approach achieved comparable results despite using a much smaller dataset.

Transformer-based models like Swin-UNet and InternImage have demonstrated impressive performance in medical image segmentation tasks due to their ability to capture global contextual information through self-attention mechanisms [22,74]. However, transformer architectures typically have higher model complexity due to extensive self-attention operations and large parameter counts, which can result in increased computational demands compared to traditional CNNs [75]. In contrast, our proposed CNN-based DRU-Net maintains competitive segmentation performance with relatively lower computational requirements, potentially making it more suitable for deployment in resource-constrained clinical environments.

Similar to H2G-Net, our proposed model, DRU-Net, also utilizes a cascaded design with two stages of PWC and refinement, and has achieved comparable results [23]. Although H2G-Net uses a lightweight PWC and a relatively heavier U-Net for refinement, our architecture—DRU-Net—demonstrated better performance when using a heavier feature extractor (PWC) combined with a lightweight U-Net. This architectural choice is particularly beneficial in scenarios with limited training data. In such cases, placing the model’s capacity earlier in the pipeline allows it to capture more discriminative and generalizable features during the initial extraction stage, while a simpler refinement network, like a lightweight U-Net, helps to avoid overfitting during the later stages. This balance ensures that the network focuses on learning robust features without excessive parameter overhead in the refinement phase. Pedersen et al. introduced a balancing technique to ensure equitable representation of available categories [23]. This helps minimize bias toward specific tissue types or tumor characteristics.

In this study, we also encountered some challenges due to the significant class imbalance between the patches derived from the WTA approach. Addressing the resultant low precision, a comprehensive strategy was implemented to improve model accuracy. Key interventions included resampling techniques, both under- and over-sampling, as well as the incorporation of focal loss, which specifically helps to address class imbalance by modulating the loss function to focus on harder-to-classify examples [76]. Furthermore, we explored the clustering of similar tissue types before sampling, the use of a weighted loss function, and adjustments to the decision threshold.

In the training phase of the many-shot model using PSA-derived samples, we deliberately introduced a controlled imbalance to optimize threshold settings and enhance performance. Experiments suggested that the deliberately-induced imbalance may offer improved performance compared to methods such as resampling, under-/over-sampling, focal loss, clustered tissue sampling, weighted loss functions, and threshold tuning [76]. However, this approach poses a risk of bias, requiring careful calibration and ongoing monitoring to prevent skewed results. The DRU-Net model’s performance was validated externally, trained on the NLCB dataset and tested on 20 slides from the HULC cohort.

The decrease in performance after fine-tuning the U-Net layers of the H2G-Net may be due to the relatively small number of annotated WSIs available in our study. Conversely, the DRU-Net network’s superior performance under similar conditions suggests the efficacy of the DR-Fused network, accompanied by a relatively lightweight U-Net architecture in data-scarce scenarios.

The relatively low performance of the original H2G-Net on NSCLCs with no fine-tuning can be explained by different tissue morphology, growth pattern, and stromal invasions, which can mislead the model during inference [42,77,78,79,80,81,82,83].

To analyze the effect of the proposed U-Net refinement network, we compared Figure 3 and Figure 4. Our results indicate that refining the PWC heatmap with the suggested refinement network improved the performance of the evaluated models. However, the main strengths and weaknesses of the models compared to each other directly stem from the PWC models and the training methods used. Additionally, combining the two processes seems to improve and reduce the variance in the segmentation DSC values, indicating that the refinement models have learned to understand overall patterns and connections, leading to a better segmentation.

The difference observed in the average DSCs between the PWC models indicates that the models trained using PSA outperformed the WTA approach under limited data conditions. This was likely due to the inadequate separability of the feature distributions between tumor and non-tumor. In the WTA approach, the method involved annotating entire tumor regions, which often included patches where the feature distributions of tumor and non-tumor tissues overlapped significantly. This overlap reduced the separability and weakened the discriminatory power of the classification models trained using this approach. Consequently, the distinction between tumor and non-tumor features in these patches became less pronounced, leading to potential misclassifications.

The PSA method adopted a more selective approach by targeting patches for annotation based on their discriminative morphology. By focusing on patches where tumor and non-tumor features were clearly distinguishable, PSA enhanced the model’s ability to accurately classify these features. This selective annotation process effectively increased the inter-class variance while reducing the intra-class variance, thus significantly improving the overall performance of the classification models in distinguishing between tumor and non-tumor tissues under conditions of limited data. In the WTA approach, the mentioned inseparable feature distribution affected the loss function negatively, resulting in lower accuracy. This was most likely rooted in the fact that the tumor regions also include other cell types than the invasive epithelial cells. By using histopathological knowledge for selecting areas with the most relevant features in PSA, the variation in the features between the two classes could be increased.

It should, nonetheless, be noted that in our case, the PSA and WTA methods were applied to different datasets. Therefore, the observed performance differences do not constitute a statistical comparison, and no definitive claims can be made about the superiority of one approach over the other.

Our study indicates that employing few-shot learning in conjunction with a clustering approach can achieve accuracy levels comparable to methods reliant on extensive datasets, potentially mitigating the need for large-scale data collection. The few-shot learning approach can be beneficial when there is a high degree of similarity within each class of tissue types and a clear distinction between the classes in the feature space [84].

One of the novel techniques presented here was utilizing an evolutionary optimization technique to determine the optimal number of clusters (classes) to minimize intra-cluster variance and maximize inter-cluster variance prior to few-shot training. This method optimally configures clusters to reflect the most coherent and meaningful class structures, which is crucial when the available training data are scarce. By focusing on minimizing intra-cluster variance and minimizing inter-cluster similarity, the approach enhances the model’s ability to generalize from limited examples, a critical aspect in few-shot scenarios where the risk of overfitting is high. Evolutionary algorithms also offer adaptability and flexibility. This enables the model to effectively handle varying data types and distributions. This pre-training optimization led to more efficient training and improved model performance by grouping patches into different classes.

The qualitative assessment of our results suggests that the DRU-Net model shows limitations in accurately delineating the tumor periphery. This challenge was particularly evident in regions with fibrosis, reactive tissue, or inflammation, where the model tends to produce false-positive and false-negative segmentations. This limitation is most likely due to the limited size of the training data; with a larger dataset containing more examples of these complex regions correctly annotated, the model’s performance in these areas might be significantly improved.

A key limitation of our study is the modest size of our dataset of 97 WSIs from the HULC cohort. Generating pixel-perfect tumor outlines on WSIs is an extremely labor-intensive process and time-consuming for an expert pathologist (including review and correction), even when using semi-automated contouring tools. Under these resource constraints, expanding beyond 97 expertly whole tumor annotated slides was simply not feasible within the project timeline. We chose to create this new dataset rather than relying on existing publicly available annotated datasets because most of them focus exclusively on neoplastic cells at the pixel level, often excluding the surrounding stroma and other intermixed cell types present within the tumor region. Additionally, comparable datasets that adopt a whole-tumor region approach typically lack the resolution and accuracy required to precisely capture tumor borders and small, scattered tumor cell clusters.

Despite the limited dataset size, we observed a consistent alignment between training and validation loss curves along with a stable performance on the external test set. This suggests that the model’s performance is not merely a result of overfitting but a genuine generalization to the tested unseen data.

In the future, we suggest reducing the model size using advanced attention-focusing mechanisms and a multi-scale patch-wise classifier to better incorporate information at different scales. Employing anomaly detection algorithms might help identify reactive tissue outliers that contribute to false-positive classifications.

Although HE is the standard coloring method for the assessment of histopathology slides, the stain can vary from laboratory to laboratory. Hence, testing on non-Norwegian cohorts and from laboratories with different staining techniques can be beneficial. We searched extensively for open-access lung tumor-segmentation datasets that include tumor outlines demarcated according to the same protocol we employ, but did not identify any that match our annotation style or resolution. As a result, quantitative evaluation of segmentation generalizability beyond the NLCB and HULC cohorts remains challenging. For future work, we suggest addressing this gap with a proper dataset with multi-institutional WSI cohorts capturing a range of scanners, staining protocols, and patient demographics. After establishing the generalizability, the model should be set up for clinical validation.

Additionally, Mask R-CNN architectures are highly effective in distinguishing complex patterns that can be used for better tumor border delineation. Implementing Bayesian neural networks can potentially improve the prediction of tumor boundaries while quantifying the uncertainty of predictions. To more effectively incorporate global WSI context, methods such as Markov or conditional random fields could be integrated along with PWC or transformer architectures. Using this approach will ensure that segmented areas are not only based on local pixel values. To further improve the differentiation between the two classes, we suggest Neuro-Fuzzy Systems, maintaining the learning capabilities of neural networks while applying the reasoning capabilities of fuzzy logic. To overcome the challenge of limited data, we suggest using unsupervised domain adaptation algorithms to leverage annotated data from other histopathology source domains.

## 5. Conclusions

In conclusion, we have introduced DRU-Net for non-small cell lung cancer tumor delineation in WSIs. Our new model, which synergistically integrates truncated DenseNet201 and ResNet101V2 with a U-Net-based refinement stage, demonstrated high performance in NSCLCs over various tested methods. Our patch-wise classifier achieves superior performance through an advanced multi-lens distortion augmentation technique and an optimized PSA strategy.

## Figures and Tables

**Table 1:** Histological subtypes of non-small cell lung carcinoma cases in the NLCB and the HULC cohorts. Counts are shown with corresponding percentages. AC: Adenocarcinoma, SCC: Squamous Cell Carcinoma, NSCC: Non-small Cell Carcinomas, WSIs: Whole Slide Images.

Histological Subtype	NLCB (*n*,%)	HULC—Train (*n*, %)	HULC—Test (*n*, %)
AC	16 (38.1%)	38 (49.4%)	7 (35.0%)
SCC	15 (35.7%)	32 (41.6%)	10 (50.0%)
Other NSCC	11 (26.2%)	7 (9.1%)	3 (15.0%)
Total number of WSIs	42	77	20

**Table 2:** Methods and experiments carried out with various models on the same 20 WSIs of the test set from the HULC cohort. Abbreviations: PWC: patch-wise classifier; HULC: Haukeland University Lung Cancer; NLCB: Norwegian Lung Cancer Biobank; FSC: few-shot (with a pre-trained MobileNetV2 [60] model) + clustering; MSC: many-shot (with a pre-trained MobileNetV2 [60] model) + clustering.

	Models	Modifications	Training Dataset (s)
(I)	H2G-Net	—	—
(II)	H2G-Net	Fine-tuned PWC	HULC Cohort
(III)	H2G-Net	Fine-tuned U-Net	HULC Cohort
(IV)	H2G-Net	Fine-tuned PWC and original U-Net	HULC Cohort
(V)	DRU-Net	—	HULC Cohort
(VI)	H2G-Net	Fine-tuned PWC	NLCB
(VII)	H2G-Net	Fine-tuned PWC and U-Net	PWC trained on NLCB, U-Net trained on HULC Cohort
(VIII)	FSC	—	NLCB
(IX)	MSC	—	NLCB
(X)	DRU-Net	—	PWC trained on NLCB, U-Net trained on HULC Cohort

**Table 3:** Qualitative evaluation scoring system.

0	1	2	3	4	5
No tumor tissue in image or segmentation, or image not suitable for analysis	Completely wrong segmentation of tumor, tumor tissue not segmented	A large part of the tumor is not segmented	Most of the tumor is correctly segmented, but some false positive or false negative areas	Most of the tumor is correctly segmented, only sparse false positive or false negative areas	The whole or almost the whole tumor correctly segmented

**Table 4:** The impact of the multi-lens distortion augmentation technique using different architectures on different datasets, randomly selecting 10% of the training data. Pairwise tests were performed using Wilcoxon signed-rank tests. The augmentation design with the highest F_1_-scores row-wise are highlighted in bold.

		F_1_-Score		
Model	Dataset	W/O Aug	W/ Aug	*p*-Value
DenseNet121	MNIST	0.9893	**0.9894**	0.2311
DenseNet121	Fashion-MNIST	0.9043	**0.9208**	<0.001
DenseNet121	CIFAR-10	0.8086	**0.8235**	<0.001
DenseNet121	CIFAR-100	0.5199	**0.5581**	0.0502
H2G-Net	NLCB	0.8299	**0.8341**	0.0701
DRU-Net	NLCB	0.8868	**0.9025**	0.0241

**Table 5:** Comparison of different backbone architectures for patch-wise classification of lung cancer tissue using the many-shot method. The best-performing architecture per metric is highlighted in bold. Abbreviations: DR: fusion of DenseNet201 (D) and ResNet101V2 (R).

Architecture	F_1_-Score	Precision	Recall
VGG19 [67]	0.87	0.86	0.87
ResNet101V2 [37]	0.89	0.89	0.89
MobileNetV2 [60]	0.86	0.86	0.86
EfficientNetV2 [68]	0.89	0.89	0.89
InceptionV3 [69]	0.90	0.89	0.91
DenseNet201 [36]	0.91	0.91	0.91
Proposed DR-Fused	**0.94**	**0.94**	**0.93**

**Table 6:** Computational complexity comparison between different backbone architectures. Metrics are reported as total FLOPs and number of parameters. The percentage increase relative to MobileNetV2 is also reported.

Architecture	FLOPs (M)	Params (M)	ΔFLOPs (%)	ΔParams (%)
DR-Fused	11,105.27	13.18	1712.42	483.02
VGG19 [67]	39,276.93	139.58	6310.14	6074.55
ResNet101V2 [37]	14,430.04	42.63	2255.04	1785.86
MobileNetV2 [60]	612.73	2.26	0.00	0.00
EfficientNetV2 [68]	1455.32	5.92	137.51	161.97
InceptionV3 [69]	5693.36	21.81	829.18	864.67
DenseNet201 [36]	8631.68	18.33	1308.72	710.68

## Data Availability

The datasets generated and/or analysed during the current study are not publicly available due to the sensitive nature of personal medical data from patients who may still be alive, but might be available from Associate Professor Hanne Sorger upon request, on a mutual collaborative basis.

## Supplementary Materials

The following supporting information can be downloaded at https://www.mdpi.com/article/10.3390/jimaging11050166/s1. Reference [85] is cited in the supplementary materials. PDF S1: Supplementary Information for Segmentation of Non-Small Cell Lung carcinomas: Introducing DRU-Net and Multi-Lens Distortion. This file contains the following: Methods that were tested to address the class imbalance in the data; Details on the alternative methods that were used for comparison against the proposed method including H2G-Net, few-shot learning, and clustering techniques; Explaining the feature distribution challenges and the deliberately induced data imbalance. The source code is made openly available https://github.com/AICAN-Research/DRU-Net (accessed on 29 April 2024).
