# Supplementary material for: Segmentation of Non-Small Cell Lung Carcinomas: Introducing DRU-Net and Multi-Lens Distortion
Source: J Imaging. 2025 May 20;11(5):166. doi: 10.3390/jimaging11050166 (PMC12112506; doi:10.3390/jimaging11050166)
Supplement: Supplementary file 1 [file jimaging-11-00166-s001.zip › jimaging-3582277-supplementary.pdf]

Supplementary Information for Segmentation of Non-Small Cell Lung carcinomas: Introducing DRU-Net and Multi-Lens Distortion

Methods that were tested for solving the class imbalance in the data

In the over-sampling approach, instances from the under-represented class were regenerated during each epoch's data generation to increase their presence in the dataset. In the under-sampling approach, a certain number of patches from the over-represented class were randomly selected during each epoch and deleted to reduce their presence in the dataset. Sampling from clustered tissue types was done by applying K-means clustering on the downsampled WSIs (downsampling ratio: 1/32) to group tissue types based on their feature similarities. The pretrained ResNet50 on ImageNet was used for extracting features prior to clustering. This grouping was performed to ensure that each cluster represents a distinct tissue type category. On top of the clustered image, oversampling was applied to ensure equal flow of tumor/non-tumor patches during data generation.

To conduct a weighted loss function in TensorFlow, class weights were dynamically calculated and adjusted during training at each step by calculating the class instance frequencies. The updated weights were then applied to the loss function by assigning the class weights. Focal loss was applied by using TensorFlow library, which provides a ready-to-use implementation of categorical focal crossentropy. Threshold tuning was carried out by monitoring the F_1-score for different threshold values on the validation set. Utilizing this metric, we identified and established a new optimal threshold value for the test data.

Compared methods

H2G-Net

H2G-Net is a cascaded CNN designed for segmenting breast cancer regions from gigapixel histopathological images[1]. It employs a detection stage with a patch-wise method and a refinement stage using a modified pretrained U-Net. The pretrained MobileNetV2 from H2G-Net was used for the original comparison and as a base model for transfer-learning[1,2]. Data were divided into two classes, tumor and non-tumor, and models were trained to predict one class for each patch (tile). With the original top classifier removed and replaced with two dense layers, all the trainable layers of the model were retrained.

Few-Shot Learning

In the few-shot learning approach, after taking images from the selected areas, an optimizing algorithm was utilized to find the optimum number of classes. We used a novel approach to optimize image clustering using a genetic algorithm (GA) combined with deep learning features extracted via a pre-trained MobileNetV2 model[2]. This methodology seeks to dynamically determine the optimal number of clusters that maximize intra-cluster similarity and minimize inter-cluster similarity among a given set of images. Since the goal was to segment the tumor regardless of its type and class, during the inference, all the classes were only labeled as tumor, and the rest as non-tumor. We used two methods, first a matching Network approach that involves creating a model that learns to match a small set of labeled examples (support set) to a new example (query). This is achieved through an embedding model and an attention mechanism. The embedding model transforms both the support set and the query images into a high-dimensional feature space. In our code, we've utilized a CNN for this purpose, which is typical for image-based tasks and we have also integrated an attention mechanism in the network. In the second approach, we used transfer learning with a pretrained MobileNetV2[2].

Clustering:

Gradient extraction:

Gradients were used for two purposes, first, to extract important features from the image that can lead to an approximation of tumor area in many cases, and second, to remove cartilage from the final mask. This is due to the extremely smooth surface of the cartilage. To acquire the gradients, we used the TensorFlow image gradients function, which returns a pair of tensors (dy, dx) holding the vertical and horizontal image gradients, they are then used to calculate the magnitude and the direction of the gradients. A different magnification level was used for each purpose (x10 for tumor approximation and x20 for identifying the cartilage).

Superpixels

We first applied the SLIC (Simple Linear Iterative Clustering)[3] algorithm to generate clustered regions (superpixels) from the image. This algorithm generates the superpixels by clustering the original pixels based on their color similarity and proximity in the image plane. Then the average color of the original pixels inside each superpixel is assigned to that superpixel. Assigning the center color was avoided due to the nature of the Histopathological images.

Clustering refinement:

Pre-segmentation analysis results (extracted gradients and superpixels), along with color channels and the PWC's probability result were used in k-means clustering. Labels were assigned automatically by assigning the overlapped regions with the highest PWC heatmap as Tumor. After selecting the centers, the algorithm starts to refine the centers and consequently the clusters. A post-clustering process is then applied to the result, executing class reassignment and median blurring that produces the final mask (Figure S1).

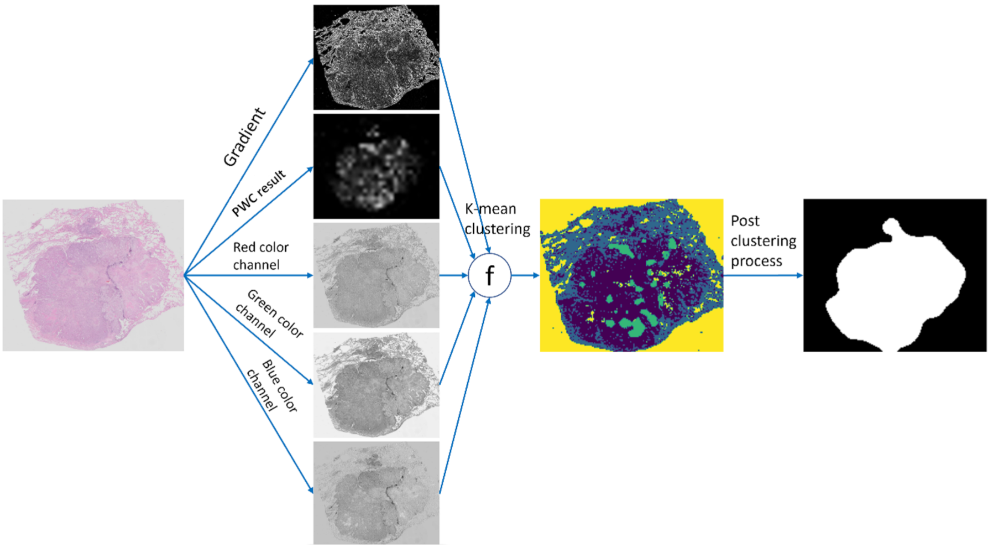

Supplementary Figure S1: Illustration of the clustering refinement technique.

Explaining the feature distribution challenges and the deliberately-induced data imbalance

Considering that the PWC is a binary classifier, it classifies each received patch solely based on its features, as either tumor or non-tumor, without considering surrounding or global information initially. This context is incorporated later during the refinement stage. Define X as a one-dimensional target space of a mapping from our N-dimensional feature space, where each point $x \in X$ represents the tissue features scaled from 0 (healthiest tissue) to 1 (tumor tissue with the highest occurrence probability), as shown in Figure S2. This mapping is neither injective nor surjective.

Define $T \subset X$ as the set of tumor-labeled features, and $H \subset X$ as the set of non-tumor-labeled features. Due to the complexity of the features, T and H commonly overlap. The threshold θ determines the classification result: if $x > \theta$, then x is classified as tumor; otherwise, as non-tumor. Misclassification of near-tumor features as tumor leads to false positives.

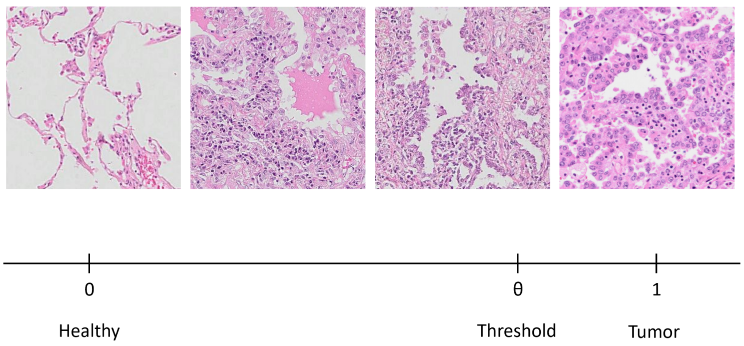

Supplementary Figure S2: Presentation of sample patches on a feature axis.

There exists $H' \subset H$ where $H' = \{x \in H \mid x > \theta\}$ and the loss function for N samples is given by:

$$L(Y, \hat{Y}) = -\frac{1}{N} \sum_{i=1}^{N} [y_{i,0} \log(\hat{y}_{i,0}) + y_{i,1} \log(\hat{y}_{i,1})]$$

where Y and $\hat{Y}$ are matrices of true and predicted labels, respectively.

Adjustments to θ and the decision function aim to reduce false positives by better distinguishing non-tumor features close to θ. By introducing more samples from H' to recalibrating θ we reduce misclassifications of H' as tumor, the classifier effectively lowers the $y_{i,1} \log(\hat{y}_{i,1})$ contributions from H' in the loss function, leading to a reduced rate of false positives. This modification to the loss function allows for a more accurate distinction between tumor and non-tumor tissues, enhancing overall classifier performance.

References

1. Pedersen, A. *et al.* H2G-Net: A multi-resolution refinement approach for segmentation of breast cancer region in gigapixel histopathological images. *Front. Medicine* 9, 971873 (2022).

2. Sandler, M., Howard, A., Zhu, M., Zhmoginov, A. & Chen, L.-C. MobileNetV2: Inverted Residuals and Linear Bottlenecks.

3. Achanta, R. *et al.* Slic superpixels. (2010). *In Proceedings of the IEEE conference on computer vision and pattern recognition*, 4510–4520 (2018).
